# Telework Arrangements and Physician Burnout in the Veterans Health Administration

**DOI:** 10.1001/jamanetworkopen.2023.40144

**Published:** 2023-10-27

**Authors:** Lucinda B. Leung, Caroline K. Yoo, Danielle E. Rose, Nicholas J. Jackson, Susan E. Stockdale, Eric A. Apaydin

**Affiliations:** 1 Center for the Study of Healthcare Innovation, Implementation & Policy, Veterans Affairs Greater Los Angeles Healthcare System, Los Angeles, California; 2 Division of General Internal Medicine-Health Services Research, David Geffen School of Medicine, University of California, Los Angeles; 3 Department of Psychiatry and Biobehavioral Sciences, University of California, Los Angeles; 4 RAND Corporation, Santa Monica, California

## Abstract

This survey study of physicians in the Veterans Health Administration examines the association of burnout with various telework arrangements.

## Introduction

Many physicians were allowed to deliver telemedicine remotely from home during the early COVID-19 pandemic, but few still telework.^1^ It is unclear whether telework can mitigate physician burnout,^2^ as recent studies have included fewer than 1000 individuals.^3^ This national study aimed to examine whether physician burnout was associated with telework within the Veterans Health Administration (VA).

## Methods

In this survey study, we analyzed data from physicians completing an annual electronic survey of all VA employees across 140 health care systems in 2020, 2021, and 2022 (mean response rate, 69%). The outcome was a dichotomized composite measure of 2 Maslach Burnout Inventory statements.^4^ Physicians rated 2 statements—“I feel burned out from my work” and “I worry that this job is hardening me emotionally”—as occurring once a week or more, on a scale from never to every day. Exposure variables were no telework by choice, unable to telework (cannot perform duties from home), unapproved to telework (can perform duties from home), part-time telework, or full-time telework. Using regression models, we examined physician burnout and telework arrangements, adjusting for survey year, physician characteristics (eg, age, gender, race and ethnicity, employment duration), and composite health care system complexity (eg, patient case mix, rurality). We conducted subgroup analyses by physician specialty and gender. This study was considered non–human participants research by the VA Greater Los Angeles Healthcare System institutional review board and was therefore exempt from the requirement for informed consent. This study followed American Association for Public Opinion Research (AAPOR) reporting guidelines.

Analyses were conducted with Stata version 17 (StataCorp). The threshold for statistical significance was α < .05 in 2-sided tests.

## Results

A total of 44 132 VA physicians responded, including 9475 primary care, 5577 psychiatry, and 9813 surgery, anesthesia, and emergency physicians. Among respondents, 23 414 (53%) were aged 50 years or older, 24 818 (56%) were men, and 25 746 (58%) were White. Averaged across 3 years, 35% of physicians reported burnout (Figure), which increased over time (2020, 29%; 2021, 36%; 2022, 39%) and was highest in primary care (52%) and psychiatry (41%). More than half did not have telework arrangements, among which 12% chose not to, 33% were unable to, and 11% were unapproved to telework. Adjusted odds of burnout were 57% (95% CI, 37%-80%) higher for physicians who were unapproved to telework, compared with those who were full-time telework (*P* < .001). Multivariable models showed 43.2% (95% CI, 41.2%-45.3%) vs 33.4% (95% CI, 30.7%-36.0%) of all physicians, 51.8% (95% CI, 47.3%) vs 43.2% (95% CI, 39.2%-47.2%) of psychiatrists, and 35.2% (95% CI, 32.7%-37.6%) vs 26.1% (95% CI, 21.4%-30.7%) of other physicians reported burnout when telework was unapproved vs full-time (Table). Lower burnout was seen among primary care physicians with greater ability to telework: 47% (95% CI, 43%-51%) for full-time, 52% (95% CI, 50%-55%) for part-time, and 61% (95% CI, 58%-65%) for physicians with telework unapproved. This association was observed across genders (men: 31% [95% CI, 27%-34%] vs 40% [95% CI, 37%-42%]; women: 36% [95% CI, 32%-40%] vs 47% [95% CI, 45%-49%]). No association was seen among surgeons, anesthesiologists, and emergency physicians, who reported similar rates of burnout and being unable to telework. Other telework arrangements, such as telework frequency and choice to not telework, had no association with physician burnout.

**Figure.**
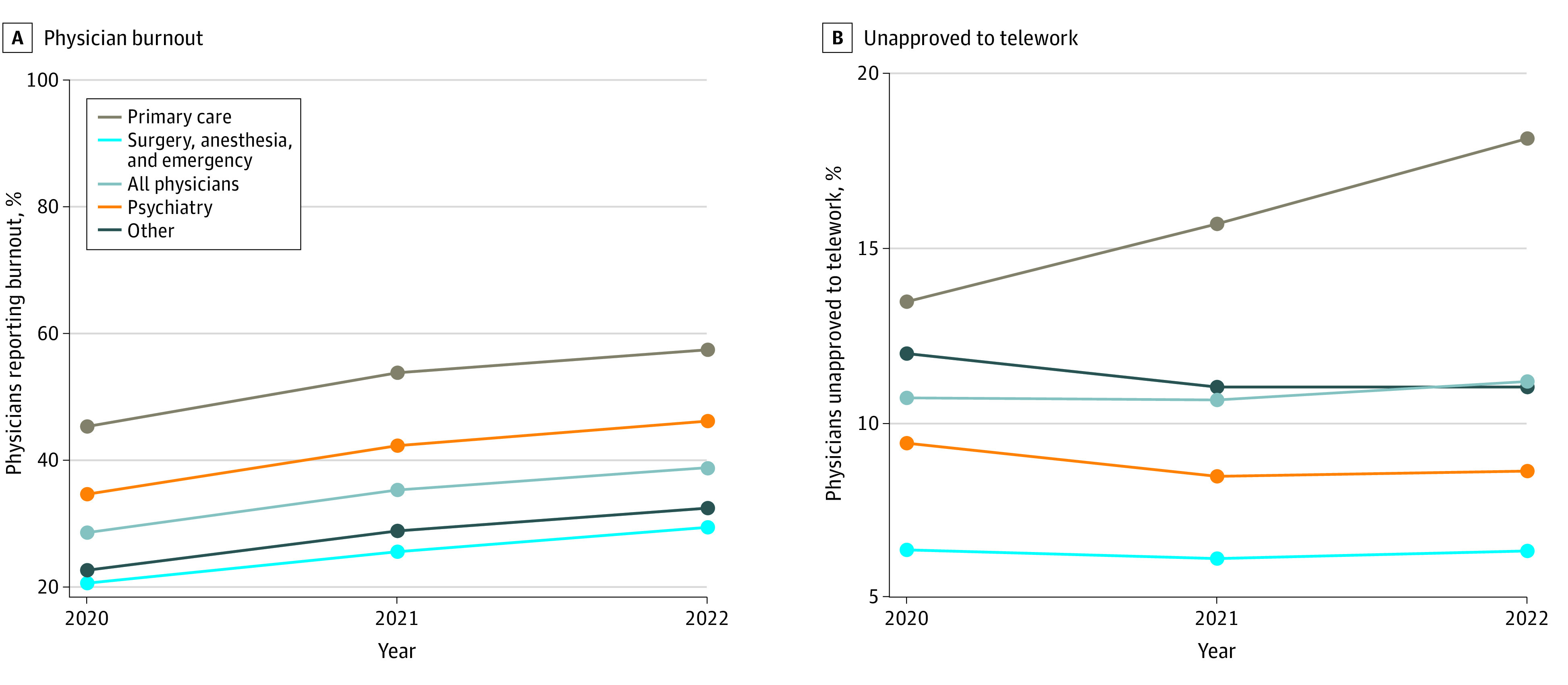
Physician Burnout Unapproved Telework

**Table.** Adjusted Percentages of Physician Burnout by Telework Arrangement and Specialty, 2020-2022^a^

Telework arrangement^b^	Physicians reporting burnout^c^									
	All physicians, % (95% CI) (n = 44 132)	*P* value	Primary care, % (95% CI) (n = 9475)	*P *value^d^	Psychiatry, % (95% CI) (n = 5577)	*P *value	Surgery/anesthesia/emergency, % (95% CI) (n = 9813)	*P *value	Other, % (95% CI) (n = 19 267)	*P *value
No telework, choice	31.1 (29.3-32.9)	.15	47.1 (43.3-50.8)	.91	36.5 (31.4-41.5)	.03	23.4 (20.1-26.7)	.53	25.6 (23.1-28.0)	.84
No telework, unable	32.1 (30.8-33.3)	.33	50.0 (47.2-52.8)	.21	35.1 (30.8-39.5)	.006	23.3 (21.6-25.1)	.50	26.8 (25.4-28.3)	.76
No telework, unapproved	43.2 (41.2-45.3)	<.001	61.3 (57.6-65.1)	<.001	51.8 (47.3-56.3)	.004	38.2 (34.0-42.3)	.17	35.2 (32.7-37.6)	<.001
Telework, part-time	34.6 (33.2-36.0)	.29	52.4 (50.0-54.7)	.03	41.0 (38.2-43.9)	.33	27.0 (24.9-29.0)	.91	28.0 (26.2-29.7)	.44
Telework, full-time	33.4 (30.7-36.0)	[Reference]	46.7 (41.2-52.2)	[Reference]	43.2 (39.2-47.2)	[Reference]	27.7 (14.7-40.6)	[Reference]	26.1 (21.4-30.7)	[Reference]

^a^ VA All Employee Surveys (AES) were administered on the following dates: September 14 to October 5, 2020; June 7 to June 28, 2021; June 6 to June 28, 2022.

^b^ The question on telework in the AES was, “How often do you telework?” Exact responses included: I do not telework because I choose not to; I do not telework because I cannot perform my job duties from home; I do not telework because I have not been approved to do so even though I could perform my job duties from home; I telework less than 1 day per week; I telework 1 to 2 days per week; I telework 3 to 4 days per week; and I telework 5 days per week.

^c^ Based on multivariable logistic regression models, adjusting for survey year, physician characteristics (eg, age, gender, race and ethnicity), composite health care system complexity (eg, patient case mix, rurality), and clustering for standard errors by VA health care system.

^d^ Statistical testing is based on odds ratios from regression models. Full-time telework was the reference category.

## Discussion

VA physician burnout continued to increase during the COVID-19 pandemic, and was highest among primary care and psychiatry specialties. Telework was available for many but not all VA physicians. We observed a statistically significant association between burnout and telework not being approved across all specialties, with the exception of surgery, anesthesiology, and emergency specialists. Telework has been linked to lower burnout, as well as higher autonomy and engagement, which are also tied to lower burnout.^5^ Although the White House has called for government employees to return to in-person work, evidence suggests that teleworkers are less likely to voluntarily quit.^6^ If flexible telework arrangements are associated with lower physician burnout, they have the potential to improve job retention and, in turn, patient care quality.^4^ To our knowledge, this study is the first to examine telework and burnout over time among US physicians. However, our survey design does not permit causal inference. We did not have information on potential confounders, like physician panel size or complexity. Further study is needed to understand circumstances whereby physicians are approved or unapproved to telework and whether telework can be an effective intervention to reduce physician burnout.

## Notes

Supplement.
Data Sharing Statement

## References

[1] US Bureau of Labor Statistics. Labor Force Statistics from the Current Population Survey: Effects of the coronavirus COVID-19 pandemic (CPS). Updated November 2, 2022. Accessed July 29, 2023. https://www.bls.gov/cps/effects-of-the-coronavirus-covid-19-pandemic.htm

[2] Shanafelt TD, West CP, Dyrbye LN, . Changes in burnout and satisfaction with work-life integration in physicians during the first 2 years of the COVID-19 pandemic. Mayo Clin Proc. 2022;97(12):2248-2258. doi:10.1016/j.mayocp.2022.09.002 36229269 PMC9472795

[3] Garavand A, Jalali S, Hajipour Talebi A, Sabahi A. Advantages and disadvantages of teleworking in healthcare institutions during COVID-19: a systematic review. Inform Med Unlocked. 2022;34:101119. doi:10.1016/j.imu.2022.101119 36373130 PMC9637285

[4] National Academies of Sciences, Engineering, and Medicine. Taking Action Against Clinician Burnout: A Systems Approach to Professional Well-Being. National Academies Press; 2019. 31940160

[5] Sardeshmukh SR, Sharma D, Golden TD. Impact of telework on exhaustion and job engagement: a job demands and job resources model. New Technol Work Employ. 2012;27(3):193-207. doi:10.1111/j.1468-005X.2012.00284.x

[6] Choi S. Flexible work arrangements and employee retention: a longitudinal analysis of the federal workforces. Public Pers Manage. 2020;49(3):470-495. doi:10.1177/0091026019886340

