# Supplementary material for: Telework Arrangements and Physician Burnout in the Veterans Health Administration
Source: JAMA Netw Open. 2023 Oct 27;6(10):e2340144. doi: 10.1001/jamanetworkopen.2023.40144 (PMC10611990; doi:10.1001/jamanetworkopen.2023.40144)
Supplement: Supplement. — Data Sharing Statement [file jamanetwopen-e2340144-s001.pdf]

Data Sharing Statement

Leung. Telework Arrangements and Physician Burnout in the Veterans Health Administration. *JAMA Netw Open*. Published October 27, 2023. doi:10.1001/jamanetworkopen.2023.40144

Data
Data available: No

Additional Information
Explanation for why data not available: The dataset used in this study cannot be shared publicly because it is 3rd party data from the VHA National Center of Organization Development (NCOD). To gain access to this data, interested researchers must complete a data use agreement with NCOD; their contact information is Scott.Moore@va.gov.
